# Measuring Changes in Social Skills Throughout an Intervention Program for Children with ASD, Contributions from Polar Coordinate Analysis

**DOI:** 10.1007/s10803-022-05496-0

**Published:** 2022-03-12

**Authors:** Carlota Alcover, M. Ángeles Mairena, Jairo Rodríguez-Medina, Marcela Mezzatesta, Gemma Balañá, Neus Elias, Maria Elias, Eulàlia Arias-Pujol

**Affiliations:** 1 FPCEE Blanquerna, Ramon Llull University, Barcelona, Spain; 2 Mental Health Department, Multidisciplinary Unit of Autism Spectrum Disorder (UnimTEA), Sant Joan de Deu Hospital, Barcelona, Spain; 3 Department of Methods of Research and Diagnosis in Education I, Faculty of Education, Universidad Nacional de Educación a Distancia (UNED), Madrid, Spain

**Keywords:** Polar coordinate analysis, Autism spectrum disorders, Social skills

## Abstract

The demand of social skills interventions for people with ASD has grown in recent years. The main goal of this research was to study social skills: “responding to interaction” and “initiating interaction”, and to capture whether there were differences between an initial and a final session in a program for children with ASD. Additionally, we aimed to compare social skills patterns according to the VIQ level. The sample (N = 20) was divided into 2 subgroups depending on whether the VIQ was > 90 or < 90. We employed a mixed methods approach based on a systematic observation of social behaviors. The observational design was nomothetic, follow-up, and multidimensional. Once we confirmed inter-observer reliability for the ad hoc observational instrument we performed descriptive statistics and polar coordinate analysis using LINCE software. The results show high intragroup and intergroup variability. In general, participants with VIQ < 90 showed a better improvement in responding to interaction, whereas participants with VIQ > 90 showed more complex patterns to initiate interactions. The polar coordinate technique was useful for detecting significant relationships between autism’s social micro-behaviors. Results and information obtained through observational methodology could allow professionals to understand communication and interaction of participants.

**Supplementary Information:**

The online version contains supplementary material available at 10.1007/s10803-022-05496-0.

## Introduction

Autism Spectrum Disorder (ASD) is a neurodevelopmental disorder characterized by alteration in social communication and social interaction, along with restricted and repetitive patterns, behaviors, interests, and activities. Impairment in social competence may cause significant problems in daily life, such as difficulties in social reciprocity, abnormality in eye contact and alteration in nonverbal communication (American Psychiatric Association, 2013, 2020). Social skills deficits may include the following difficulties: challenges with entering, sustaining and exiting interactions; difficulty attending to, understanding, and using nonverbal and verbal social cues (eye contact, facial expressions and gestures (Attwod et al., 1988); difficulty in understanding the social rules of their context; difficulties to understand the other’s intentions, perspective and to interpret the beliefs of others; difficulty in problem solving and problems with participating in leisure activities and in free time play with others (Hyman et al., 2020). McMahon et al. (2013) commented that social response is a difficult skill due to the need to be conscious of the other’s speech and thoughts in order to be connected to the conversation. As a consequence of these alterations, difficulties in social interactions may interfere in their relations. Moreover, social impairments in youth with ASD do not tend to improve merely with development, but rather may become more pronounced during adolescence when the social demands exceed the social skills (Gates et al., 2018).

### Intervention Programs

In order to target these nuclear difficulties, social skills interventions have demonstrated to be useful to improve social competence and might consequently improve comorbid affective symptomatology (Gates et al., 2018). Commonly, programs include domains of verbal and nonverbal communication, social interaction and problem-solving strategies. There are several recommended social skills interventions (National Institute for Health & Care Excellence, 2013). The most common is social skills groups, which tend to be used in school-aged children and adolescents (Chester et al., 2019). Peer mentoring/training, is one of the most effective interventions in social skills and is regularly applied in preschool and school-age children in classrooms (Bohlander et al., 2012). Video modeling has been shown to be effective to improve social and communication skills in children and adolescents with ASD (Buggey, 2009). Like peer mentoring, video modeling has proved that skills tend to be generalized and maintained (Tierney et al., 2014). Social narrative, social stories and picture books are widely used as tools to complement social skills training (Golzari et al., 2015; Reichow & Wolkmar, 2010). There are many published social skills programs which have been used with children with ASD, such as Think Social! (Garcia Winner, 2007), Social STAR (Arick et al., 2004), Navigating the Social World (McAfees, 2009) and Social Adjustment Enhancement Intervention (Solomon et al., 2004). However, further research is needed in order to support the use of social skills programs.

### Factors that Interfere with Social Skills Interventions

Research has analyzed the effect of certain factors on the effectiveness of social competence interventions, such as age (Hong et al., 2018), sex (McVey et al., 2017), severity, comorbidity or IQ. However, these results are not yet consistent (McMahon et al., 2013; McMahon et al., 2013). For instance, whereas some studies have found higher effectiveness in participants with a normal range of IQ, other studies do not confirm this relationship (Gates et al., 2018). Interestingly, specific factors may interfere with each other. For instance, age, intelligence quotient (IQ) or gender, could interfere with the presence of comorbidities (Guerrera et al., 2019). Regarding age, adolescents present more difficulties related to anxiety or depression compared with children in the spectrum (Bellini, 2006; Mensi et al., 2013). In relation to IQ, certain studies report a relationship between IQ and comorbidity (Bölte et al., 1999) but other researches do not confirm this finding (Avni et al., 2018). In terms of gender and comorbidity in ASD, no relationship has been found in recent studies (Margari et al., 2019) but more studies are needed. In fact, further research should clarify the relationship between specific factors and response to social skills interventions. Studies should explore the relationship between Verbal IQ (Verbal Comprehension), which has an important role in social communication, and performance in social skills programs.

### The Present Proposal

Based on all the previous literature revised, our proposal is based on a phrase from Creswell and Plano Clark (2011, p. 7) that we especially value: "There are three ways in which mixing occurs: merging or converging the two datasets by actually bringing them together, connecting the two datasets by having one build on the other, or embedding one dataset within the other so that one type of data provides a supportive role for the other dataset". This mixing, applied from a literal and broader perspective, constitutes a central point of support for rethinking the *quantitizing*. On one hand, from literality, “connecting the two datasets by having one build on the other” will imply that one database—which is qualitative in nature, in our paper social skills group sessions—can give rise to another through its transformation. This transformation must ensure the maintenance of its informative quality, although the appearance is modified. On the other hand, from a broader perspective, the *connecting* allows the alternation of QUAL-QUAN-QUAL stages, which legitimizes the generic approach of mixed *methods,* while a total integration between qualitative and quantitative elements is achieved (Anguera et al., 2020). With this rethinking we can ensure an idoneous way to materialize in this paper.

In this work, focused on procedural aspects, we propose an observational methodology considered itself as a mixed method (Anguera & Hernández-Mendo, 2016; Anguera et al., 2017), that implies integration ways (quantitizing) between qualitative and quantitative elements. The proposal of quantizing begins with a systematic observation of ASD children, and implies qualitative records (QUAL step); from theoretical framework and empirical expertise we built an observation instrument with a specific structure that allows to record from sequence parameter, and following a matrix codes format, that we will analyze quantitatively (QUAN step); the results will interpret coming back to initial problem (QUAL step) (Anguera et al., 2020).

Specifically, it has been suggested that observational methodology can complement other methodologies, allowing to analyze spontaneous conducts that take place in natural situations (Anguera, 2003; Portell et al., 2015) and behaviors in psychotherapy settings (Arias-Pujol et al., 2015). This methodology also allows to detect patterns in co-therapy work with a group of siblings of children with autism (Venturella et al., 2019), to analyze evolution of parenting skills during a behavior parent training with ASD children (Parladé et al., 2019).

Considering all previous literature, the main objective of this research is to provide more scientific evidence about new mixed methodologies, measuring possible changes in social behavior in children with autism. Specifically, this research aims to (a) observe changes between the second and the last session of a social skills intervention program for children with ASD, through Polar

Coordinate Analysis (we were interested in studying social skills: “responding to interaction” and “initiating interaction”); (b) detect if there exist differences in children regarding the intelligence quotient, to compare social skills patterns according to the VIQ level. We hypothesized that results from Polar Coordinate Analysis would be different in session 2 and session 10, showing differences in children with high VIQ.

### Our Intervention Program

The intervention that we applied was an adaptation of the Social Skills program of the UC Davis MIND Institute (Solomon et al., 2004). This intervention has shown positive results, including an increase in responses and interaction between peers and a decrease in other vocalizations, such as talking alone (McMahon et al., 2013; McMahon et al., 2013). The main objective of the intervention group was to work with patients from an inside-out perspective, developing and practicing the abilities of empathy, recognition of their own and others’ emotions and problem resolution, conversational skills or stress regulation, among others.

Considering our community hospital context and the high demand of intervention in social skills, we adapted the original program to a brief intervention of ten sessions (Solomon et al., 2004) with two purposes: one objective was to be able to reach all patients in the ASD unit who needed this type of intervention, offering a lower waiting list and a brief intervention, compatible with the public resources of the hospital. Another purpose was to observe whether a brief intervention program (adapted to our community context) could cause initial and little changes in social behavior (Lerner & Mikami, 2012; Matthews et al., 2019).

## Method

### Design

The study was based on a quasi-experimental design with measurement before and after treatment in a group of participants. Regarding observational methodology, the design of our research was N/F/M, nomothetic (N) because several participants were observed, it consisted of follow-up (F) because an initial session (session 2) and the last session from the intervention were registered, and it was multidimensional (M) because several dimensions of the observation instrument were considered suitable (Anguera, 2011; Sánchez-Algarra & Anguera, 2013).

### Participants

According to the approval to the standard of the institutional Research Committee review board, the Ethical Committee for Clinical Research (Intern Code: PIC-04-17), and following the 2000 Helsinki declaration, participants were recruited by psychologists and psychiatrists from the Multidisciplinary Autism Spectrum Disorder Unit. All parents provided written and informed consent and informed assent were obtained from each child. Participants were informed about the location of the camera and the period of time that would be recorded.

Participants were selected through the inclusion criteria: 8–12 years old, diagnosis of Autism Spectrum Disorder verified with the Autism Diagnostic Observational Schedule-2 (ADOS-2, Lord et al., 2000), and normal range of level of Verbal Comprehension (Verbal IQ) according to standardized assessment (WISC-IV or V, Wechsler & Kaplan, 2015). Participants with severe behavioral problems, other several mental disorders such as schizophrenia, or with intellectual functioning below 70, were excluded.

During 2 years (2018, 2019), a total of 36 children were recruited to participate in the intervention sessions. Each year we recruited 18 children and paired them according to age, gender and VIQ; then each pair was randomized between the two groups using R software (R Foundation for Statistical Computing; Vienna, Austria) with a fixed random seed. Each group was formed by 7, 8 or 9 participants and conducted by a clinical psychologist, a psychiatrist and a mental-health nurse. The team had large experience in implementing social skills interventions with ASD children and received specific training to conduct this program. Besides, two master’s degree students participated in the implementation of the program. A total of four groups received the same intervention with similar conditions.

In order to code observational data, we only included 21 children (3 females and 18 males) from the total sample. This selection was based on the fact that participants of this subgroup had attended all the sessions and it was possible to codify their social behaviors through the video records. The rest of the participants (n = 15) did not attend all sessions and observational data was missing. From the final sample (n = 20), 11 participants had received the intervention in 2019 (5 children of one group; 6 of another group) and 10 participants had attended the sessions in 2018 (5 children of each group). To perform polar coordinate analyses, we included 20 participants from the sample, because data from VIQ from one participant was missing.

### Instruments

#### Diagnostic Instruments

In order to confirm the diagnoses of autism, a clinical interview based on DSM 5 criteria (APA, 2013) was done. Additionally, the ADOS-2 (Lord et al., 2000) was administered to all participants. To assess Verbal Comprehension, cognitive abilities were measured using the Wechsler intelligence scale for children and adolescents (Wechsler, 2007; Wechsler & Kaplan, 2015).

#### Recording Instrument

Sessions were recorded with two different cameras located at different angles. Cameras were positioned discreetly to avoid inconvenience to participants and followed all the ethics aspects.

#### Observational Instrument

To codify social behaviors, we reorganized and evolved an observational instrument (Alcover et al., 2019) which had been inspired by an original observational instrument of Bauminger (2002) and by the approach of social difficulties by the authors of ADOS-2 and Autism Diagnostic Interview-Revised (Lord et al., 2000; Rutter et al., 2003). Certain behaviors were not included in the analysis because the observation frequency was very low such as proximity, sharing objects, affection, talk that reflects an interest in another child's hobbies, giving help, peer or therapist imitation, idiosyncratic language and repetitive behavior. We reorganized the observational instrument in order to adjust it to the behavior of our participants (see Table 1). The final instrument had 6 dimensions. For each dimension, we built a category system, following the requirements of exhaustivity and mutual exclusivity. Dimensions and categories are shown in Table 2.

**Table 1:** Specific didactic topics of each session. Adapted from Solomon et al (2004)

Session	Didactic topic
Session 1	Presentation and empathy. We start playing different games to know each other. We introduce the concept of “empathy” and the steps, through a role playing
Session 2	Practice empathy throw role playing invented situations and examples of their real situations
Session 3	Recognizing our own and others’ emotions. Explain the concept and perform different games to understand and interiorized the emotions
Session 4	Practice emotional recognition
Session 5	Strategies to manage stress, anxiety and anger
Session 6	Practice strategies to manage stress, anxiety and anger, through role playing of real situations
Session 7	Interests and group reciprocal conversation. Talk about personal interests that have in common and differences between the group. Practice of having a conversation of topics that are not interesting
Session 8	Nonverbal communication and reciprocal conversation. Practice through role playing
Session 9	Solving social problems. Expose different real situations of problems, talk about the different solutions and possibilities to manage them. Practie through role playing
Session 10	Closing group: feelings and thoughts about the intervention group

**Table 2:** Dimensions and category systems of the social behavior observational instrument for participant

Dimensions categories (CODES)	Description	Examples
*Interaction type*		
Low-level interaction (LLI)	The child exhibits behaviors that indicate social intention, but with minimal social enactment, just to obtain something	Interactions between different participants: “It is your turn” “Pass me the token” Questions or comments related to the game
High level interaction (HLI)	The child exhibits verbal and nonverbal social behaviors that lead to an effective social process with peers. Behaviors that serve to start or maintain social interaction	“I like your t-shirt” “Do you want to play with me?” “How was your weekend?” “I want to play with you”
Negative level interaction (NLI)	Participant exhibits rude and unpleasant social behaviors	“You are a dumb” “I’m not going to let you play” Comments that include teasing or insults
*Social behavior*		
Responses to an interaction (RES)	The child responds verbally and/or nonverbally to social stimuli directed toward him/her by peers	“Which game do you want to play? Response: “I want to play Uno” “What did you do last weekend?” Response: “I went to the park”
Initiations of interaction (IN)	The child begins a new social sequence, distinguished from a continuation of a previous sequence by a change in activity	“Do you want to play with me?” “This weekend I had a problem” “My favorite animal is the lion, and yours?” “Can we change the game?”
Evitations (EV)	The child avoids any type of interaction or communication that is addressed to him/her	When someone asks to play or share something, the participant ignores or avoids the question/demand
Functional Play (FP)	The child play with another participant without talking. Some of the games do not require to speak directly	Two participants who play’three in line’ without speaking. They do not share the experience, or talk about anything, but they play together
*Verbal communication*		
Functional communication (FUNC)	The child approaches or responds to another child with an intention to fulfill his/her own needs, and with no social intention	“It’s my turn on the computer now” “Pass me the token” “It’s your turn”
Social verbal communication (SOVERC)	The child approaches another child with a social (rather than functional) intention	“Let’s play” Questions and comments related to show interest in other participants
Sharing experiences (SHAREXP)	The child talks about an experience to peers or asks them about their experiences	“What did you do over the weekend?” “I went to the mountains last summer, what did you do?”
Sharing object (OBJ)	The participant shares an object or game with another participant	The participants give a doll to another one, spontaneously or by request
Non-functional communication (NONFUNC)	Sounds, words and phrases that are not understandable or are not addressed to anyone	Sounds made during a play game but not addressed to any other participant (sing)
Verbal aggressive communication (VAGC)	The participant behaves intrusively and negatively towards his peers	“I do not want to play with you (screaming, jostling)”
*Facial expression*		
Smile (SMIL)	Participant smiles to another in an interaction, intentionally	When two participants are talking, in a social conversation or during a game, one participant smiles as an answer (combined or not with words)
*Looking*		
Not looking (NOLOOK)	Looking to another side, avoiding eye contact	When someone talks to the participant, he/she is looking to another side, avoiding eye contact
Eye contact (EC)	The child looks into the eyes of another child	When someone talks to the participant, he/she is looking at the eyes
Looking without eye contact (NOEC)	The child looks at the other child’s face or body, or child’s action, without establishing eye contact	When someone talks to the participant, he/she is looking to the other person but not to the eyes When a participant is talking, he/she is looking at the person but not to the eyes
Gestures		
Pointing gestures (POINTG)	Point your hand, arm or finger at anything to show it to another	“Move your piece here (pointing to the place)”
Emotional gestures (EMOG)	Gesture that indicates an emotion	Covering your mouth (surprise or laughter)
Conventional gestures (CONVG)	General and universal gestures, used in everyday life	Greet, raise the hand, no / yes (with your head), come, shut up, ok, etc
Affirmation or denial gestures (AFFDEN)	The child only nods his/her head for yes or shakes it for no	The participant answers a question without words, only using the head to say yes or no
Descriptive gestures (DESCG)	Gesture that is made with the arms and hands and gives us specific information and description about something	The gesture indicates the quantity, the size, the form, the length of things
Proximity (PX)	The child is near other children or game but does not play or do another activity. There is no eye contact or verbal communication	Participant reads a book and sits near two other participants that are playing cards

### Procedure

For this study, data was collected during the free playtime group (14 min) of the second and the last session using the observational instrument. We performed the analysis through two levels of response: initiation and response. We selected session 2 as pre-evaluation instead of session 1 because participants did not know each other before the intervention and the interaction during the first session was interfered by this fact.

#### Implementation of the Program

As aforementioned, our program was an adaptation of the Social Skills program of the UC Davis Mind Institute (Solomon et al., 2004). We implemented 10 sessions of 1:30 hours each. Solomon’s program is less structured than most traditional programs. It includes semi-structured instructions, positive reinforcement, motivation for social interactions, and free-time play as an opportunity to practice. Regarding the intervention, the structure of each session was: salutation with a little talk about the week and introduction of the topic (allowing participants to talk and share thoughts or problems), free time to play (were children play games in an unstructured time-lapse), didactic activity (structured part were pedagogic information and activities of social competence are performed) and closing (structured part that include joke-telling time and the optional homework, called “social experiment for the week”). The specific contents of each didactic session are detailed in Table 1. Clinicians developed strategies and activities based on theory of mind and social competence (Garcia Winner, 2007) in order to practice the didactic components.

The sample was divided in four groups in order to facilitate group intervention. Each group was formed by 7, 8 or 9 participants and each group received the same intervention with similar conditions. As aforementioned, the clinical team that conducted the groups included a clinical psychologist, a psychiatrist and a mental-health nurse with large experience in social skills interventions with ASD children. After obtaining approval from the authors (Solomon and colleagues), our clinicians were trained in the program by a member of the team who had previously collaborated with the developing team of the program at the UC MIND Institute. The clinical psychologist was the main therapist of the intervention, while the psychiatrist and the mental-health nurse were co-therapists. Additionally, two master’s degree students participated as co-therapists in order to support children in a more individual way.

In order to ensure fidelity of implementation of the intervention, before each session, clinicians had a meeting with the member of the team who had previously collaborated with the developing team of the program. In these meetings, specific instructions for each session were provided to the team and a protocol with a complete description of steps was reviewed. By the end of each session, clinicians reviewed again the specific protocol and its checklist, in order to ensure that the intervention was provided consistently and accurately as designed.

#### Data Quality Control Analysis: Inter-Observer Agreement

From the qualitative research perspective, a systematic observation was used to obtain data that we managed as a code matrix. Two psychologists who did not participate in the intervention were trained on the observation instrument in order to analyze and code data. These observers were aware of the research hypotheses, but they did not have information about the session number (session 2 or 10) or the students' IQ groups. The degree of interobserver agreement calculated with Cohen’s Kappa () ranged between 0.76 and 0.89. To obtain this value, 20% of the material was coded and the Kappa coefficient of the 2 and 10 sessions (which were randomized) was obtained of the 10% of the participants. This inter-observer agreement was conducted between the observers and the main therapist of the intervention, who had collaborated with them in the adaptation of the observational instrument. Once we had confirmed the reliability of the data, we codified 14 min during the free play activity of session 2 (as a pre-evaluation) and 10 (as a post-evaluation) of the participants that participated in all sessions and appeared in all videos, to observe differences between sessions. Social conditions of both sessions were identical. The same group of children and therapists were present and the same materials were offered to the participants.

#### Data Analysis: Mixed Methods Perspective

The software LINCE (Gabin et al., 2012) was used to codify social behaviors during the free time of the selected sessions. After the codification, the analysis strategy was developed in two stages.

First, in a descriptive level, the absolute frequencies of each of the behaviors recorded were analyzed and compared according to the groups of participants and the session (2 and 10 session).

In the second stage, *mixed methods methodology* was used in order to integrate qualitative and quantitative elements. This approach implies the integration of different types of data, which includes the transformation of quantitative data into qualitative data (Sandelowski et al., 2009), of qualitative data into quantitative data (Arias-Pujol & Anguera, 2017, 2020), or any other type of information (Onwuegbuzie & Teddlie, 2003) in a comprehensive way (“crossover”, according to Onwuegbuzie & Dickinson, 2008). We selected this methodology due to the lack of objective measures that reflect the real progress of social skills abilities. The majority of studies use questionnaires to assess the evolution of participants, but these methods tend to be subjective and incomplete (McMahon et al., 2013; McMahon et al., 2013; Moody et al., 2020). Therefore, additional methodologies that complement questionnaires have been proposed, such as social cognitive assessments and behavioral observations (Alcover et al., 2019; Solomon et al., 2004). These methodologies have demonstrated to be effective in assessing evolution and changes in social behaviors, including micro-conducts like gestures. In addition, they provide greater knowledge and specificity about social behaviors, which can offer information for professionals to improve their interventions and focus them on their participants (Alcover et al., 2019). As aforementioned, mixed methods methodology allow to transform qualitative data into quantitative data.

Specifically, in our study, we first used an observation instrument, which is a qualitative procedure. We registered categorical variables (behaviors) which were then transformed into quantitative data, in order to obtain parameters such a frequency, order and duration. Finally, the polar coordinate technique (Cochran, 1954) was applied to compare the social interactions generated in sessions 2 and 10. We selected this technique because it offers information about the relationship between a focal behavior and other conditional behaviors. Polar coordinate technique, applied by Sackett (1980) and further optimized with the genuine retrospective technique proposed by Anguera (1997), allows the reduction of data by using the Zsum statistic (Zsum = Σz/√n), where Z represents the independent values obtained from the adjusted residuals found for the respective delays of − 5 to − 1 and 1 to 5, and n represents the number of delays considered. Previous literature suggests that conduct patterns tend to get dissolved after 5 delays (Bakeman & Gottman, 1997). The sequential analysis of delays allows us to control the random effect. The goal is to identify sequences that appear in a higher frequency than expected by random. Thus, the Zsum values allow us to estimate the type of relationships established between the selected focal behavior and the other behaviors (conditioned behaviors) that constitute the instrument of observation. The type of relationship between focal and conditioned behaviors is shown qualitatively (Quadrant I, II, III or IV) and quantitatively (vector length). Quadrants indicate whether the focal and conditional conducts activate or inhibit each other. Activation involves that when a conduct occurs, another conduct is elicited. In contrast, inhibition means that one conduct might inhibit another. Vectors in Quadrant I indicate mutual excitation between focal and conditional behavior. Quadrant II informs about inhibitory focal behavior and excitatory conditional behavior. Vectors in Quadrant III indicate mutual inhibition between focal and conditional behavior. Quadrant IV informs about excitatory focal behavior and inhibitory conditional behavior.

## Results

### Descriptive Statistics of Participants

The mean age of the participants was 9.52 y.o. (median = 10, PC25 = 8.5 and PC75 = 10.5) and the mean of Verbal Comprehension Index from the WISC-IV or V scale (Wechsler, 2007) was 103.8 (median = 105, PC25 = 89 and PC75 = 117) (see Table 3).

**Table 3:** Participants demographics

*Demographics*	All participants (N =	Participants with VIQ < 90 (n =	Participants with VIQ > 90
Mean age (SD)	9.5 (1.12)	9.5 (1.17)	9.8 (1.09)
Mean verbal index quotient	103.8	84.2	112.2
Sex	18 males 3 females	5 males 1 female	12 males 2 females

A Descriptive Statistical Analysis of Session 2 and 10 from each category were performed (see Table S1). A total of 1631 behaviors in session 2 and a total of 1393 behaviors in session 10 were analyzed in 21 participants (85.7% male). In general, in session 2 (initial session), the average frequency of positive social interactions (HLI) (M = 1.14, SD = 1.39, range [0–4]) was lower than the average frequency of low intensity interactions (LLI) (M = 20.24, SD = 6.74, range [7–30]).

A general decrease in the number of social behaviors was observed in session 10, even so, an increase in the number of behaviors related to ASD nuclear difficulties such as positive social interaction (HLI; S2 = 5.65%; S10 = 12.67%) (M = 2.2, SD = 3, range [0–11]), verbal social communication, eye contact or emotional gestures was observed, as well as a decrease of the low-level interactions (LLI) (M = 14.7, SD = 5.1, range [3–23]). Finally, negative level interaction (NLI) was registered in session 10 (M = 0.4, SD = 0.7, range [0–2]).

### Outcomes Results

#### Results of Polar Coordinate Analysis

To perform these analyses, we included 20 participants from the sample, because data from VIQ from one participant was missing. Coordinate analyses were performed in two subgroups: Verbal IQ (VIQ)> 90 (n = 14) and VIQ < 90 (n = 6).

#### Focal behavior: Response to an Interaction

**Fig. 1:**
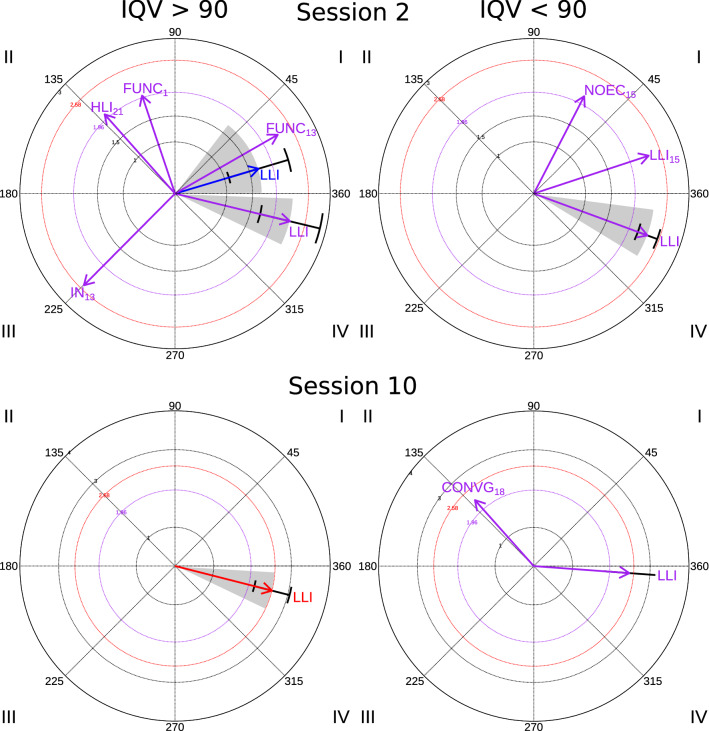
Polar coordinate results in Response to interaction as a focal behavior in sessions 2 and 10 in participants with VIQ > 90 and VIQ < 90

In session 2 (see Table 4 and Fig. 1), a significant activation relationship was observed between focal behavior response to an interaction and low-level interaction conditioned behavior, in 9 of the 14 participants with VIQ > 90. Simultaneously, low-level interaction (LLI) inhibited the response to interactions, since the vector with a significant radius representing LLI is located in quadrant IV (Quadrant IV, radius = 2.29, SD_radius_ = 0.59, angle = 346°, SD_angle_ = 8.91, p < 0.05). On the other hand, for the rest of the participants with VIQ > 90 (5/14), there was a relationship of mutual activation between response and low-level interaction (LLI), but it was not statistically significant (Quadrant I, radius = 1.69, p > 0.05). In two of these participants, functional communication (FUNC) significantly activated the response to interactions (participant 13, Quadrant I, radius = 2.29, p < 0.05; participant 1, Quadrant II, radius = 2, p < 0.05). However, while for the first participant, the response to interactions in turn activated functional communication, for the second, these responses inhibited functional communication. Finally, we observed again in one participant from VIQ > 90 group that high-level interaction (HLI) activated the response to interactions (participant 21, Quadrant II, radius = 2.05, p < 0.05) and initiations of interaction (IN) inhibited the appearance of new social responses for another (participant 13, Quadrant III, radius = 2.5, p < 0.05).

**Table 4:** Polar coordinate analysis results corresponding to response to interaction as the focal behavior

Code	Quadrant	Ratio	Radius	Angle	SD radius	SD angle	Angle range		nº participants
	*Session 2*								
	*IQV > 90*								
LLI	IV	− 0.44	2.29	346.46	0.59	8.91	334.16	357.84	9
LLI	I	0.09	1.69	16.60	0.60	21.75	0.11	52.63	5
FUNC	I	0.50	2.29	29.81	–	–	–	–	1
FUNC	II	0.95	2.00	108.58	–	–	–	–	1
HLI	II	0.75	2.05	131.60	–	–	–	–	1
IN	III	− 0.71	2.50	225.17	–	–	–	–	1

Code	Quadrant	Ratio	Radius	Angle	SD radius	SD angle	Angle range		nº participants
	*Session 10*								
	*IQV > 90*								
LLI	IV	− 0.43	2.58	345.73	0.47	8.18	334.43	356.59	5
	*IQV < 90*								
LLI	IV	− 0.08	2.47	355.81	0.66	0.23	355.64	355.81	2
CONVG	II	0.75	2.28	131.72	–	–	–	–	1

*LLI* low-level interaction, *FUNC* functional communication, *HLI* high level interaction, *IN* initiations of interaction, *NOEC* looking without eye contact, *CONVG* conventional gestures

Coinciding with the other group, we observed that low-level interaction (LLI) inhibited the response to interactions observed in 5 of the 6 participants with VIQ < 90 (Quadrant IV, radius = 2.34, SD_radius_ = 0.19, angle = 340º, SD_angle_ = 8.98, p < 0.05). Other findings obtained in the VIQ > 90 were not observed in this group, but a significant relationship of mutual activation between look without eye contact (NOEC) and response to interactions in one of the members of this group was found (participant 15, Quadrant I, radius = 2.34, p < 0.05).

In session 10, we observed again, in fewer participants, the previous relationship of significant activation between focal behavior response to an interaction and conditioned behavior low-level interaction (5 of the 14 participants with VIQ> 90) (Fig. 1) (Quadrant IV, radius = 2.58, SD_radius_ = .47, angle = 345.73°, SD_angle_ = 8.18, p < 0.01; in 2 of the 6 participants with VIQ < 90 (Quadrant IV, radius = 2.47, SD_radius_ = 0.66, angle = 355.8°, SD_angle_ = 0.23, p < 0.05).

In session 10, regarding to the VIQ < 90 group, we observed in one participant how conventional gestures (CONVG) activated responses to interactions while they inhibited these types of gestures (participant 18, Quadrant II, radius = 2.28, angle = 131.72°, p < 0.05). No other significant relationships between behaviors were observed in this subgroup.

#### Focal Behavior: Initiation of Interaction

The set of associated behaviors was significantly greater for this focal behavior than for the response to interaction.

**Fig. 2:**
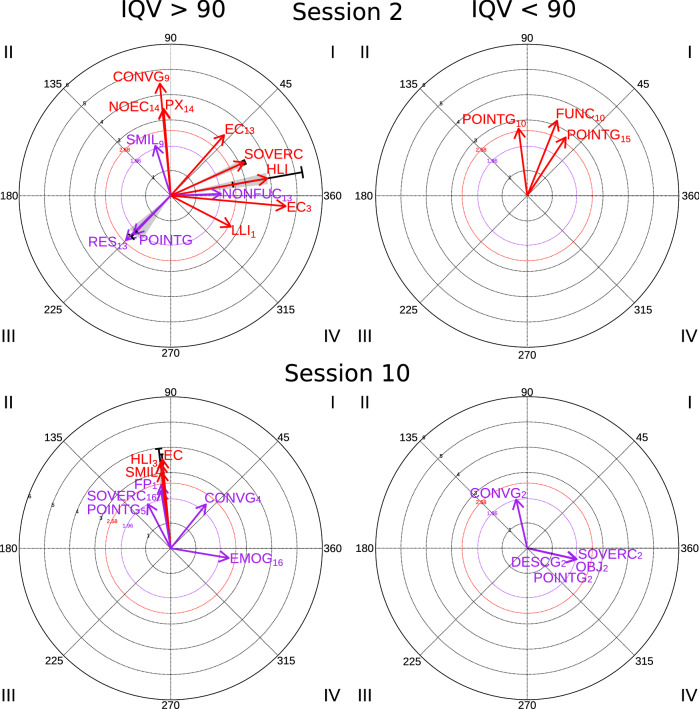
Polar coordinate results in Initiation to interaction as a focal behavior in sessions 2 and 10 in participants with VIQ > 90 and VIQ < 90

In session 2 (see Table 5 and Fig. 2), the group of participants with VIQ> 90 had a greater initiation of interactions (11 of 14 participants initiated social interactions in the initial session (M = 5.64, SD = 3.58, Range [1–12]), compared to the other group (only 3 of 6 participants, M = 2.67, SD = 2.08, Range [1–5]). In two of the participants in this group, there was a significant relationship of mutual activation between high level interaction (HLI) and focal behavior (initiation of interaction) (Quadrant I, radius = 3.90, SD_radius_ = 1.39, angle = 10.11°, SD_angle_ = 5.18, p < 0.01). Also, we observed a relationship of mutual inhibition between focal behavior (initiation of interaction) and deictic gesture behavior (POINTG) in 4 of the participants (Quadrant III, radius = 2.12, SD_radius_ = 0.15, angle = 227.98°, SD_angle_ = 8.09, p < 0.05). Mutual activation relationships between focal behavior (initiation of interaction) and verbal social communication (SOVERC) were also observed in 2 participants (participants 3 and 13: Quadrant I, radius = 3.20, SD_radius_= 0.04, angle = 24.37°, SD_angle_ = 3.94, p < 0.01) and activation of eye contact (EC) by focal behavior (initiation of interaction) (participant 3, Quadrant IV, radius = 4.57, angle = 354.75°, p < 0.01) (participant 13, Quadrant I, radius = 3.2, angle = 48.59°, p < 0.01).

**Table 5:** Polar coordinate analysis results corresponding to initiation of interaction as the focal behavior

Code	Quadrant	Ratio	Radius	Angle	SD radius	SD angle	Angle range		nº participants
	*Session 2*								
	*IQV > 90*								
POINTG	III	− 0.71	2.12	227.98	0.15	8.09	218.35	237.60	4
HLI	I	0.24	3.90	10.11	1.39	5.18	6.44	13.77	2
SOVERC	I	0.37	3.20	24.37	0.04	3.94	21.58	27.15	2
EC	IV	− 0.09	4.57	354.75	–	–	–	–	1
EC	I	0.75	3.20	48.59	–	–	–	–	1
CONVG	II	1.00	4.46	95.53	–	–	–	–	1
LLI	IV	− 0.46	2.68	332.69	–	–	–	–	1
NOEC	II	1.00	3.45	94.69	–	–	–	–	1
NONFUC	I	0.04	1.99	2.06	–	–	–	–	1
PX	II	1.00	3.42	94.73	–	–	–	–	1
RES	III	− 0.70	2.50	224.83	–	–	–	–	1
SMIL	II	0.96	2.07	107.16	–	–	–	–	1
	*IQV < 90*								
FUNC	I	0.93	3.20	68.33	–	–	–	–	1
POINTG	II	0.99	2.68	97.39	–	–	–	–	1
POINTG	I	0.83	2.77	56.59	–	–	–	–	1

Code	Quadrant	Ratio	Radius	Angle	SD radius	SD angle	Angle range		nº participants
	*Session 10*								
	*IQV > 90*								
EC	II	1.00	3.55	95.41	0.19	1.94	94.03	96.78	2
FP	II	0.99	2.55	98.29	–	–	–	–	1
SMIL	II	1.00	3.04	96.87	0.93	4.06	94.00	99.74	2
HLI	II	0.99	3.38	96.83	–	–	–	–	1
CONVG	I	0.78	2.23	51.28	–	–	–	–	1
POINTG	II	0.89	1.97	117.47	–	–	–	–	1
SOVERC	II	0.99	2.31	99.61	–	–	–	–	1
EMOG	IV	− .17	2.33	350.47	–	–	–	–	1
	*IQV < 90*								
SOVERC	IV	− 0.22	2.00	347.34	–	–	–	–	1
CONVG	II	0.98	1.98	102.79	–	–	–	–	1
DESCG	IV	− 0.22	1.98	347.21	–	–	–	–	1
POINTG	IV	− 0.22	1.99	347.28	–	–	–	–	1
OBJ	IV	− 0.22	2.01	347.40	–	–	–	–	1

*POINTG* pointing gestures, *HLI* high level interaction, *SOVERC* social verbal communication, *EC* eye contact, *CONVG* conventional gestures, *LLI* low-level interaction, *NOEC* looking without eye contact, *NONFUC* non-functional communication, *PX* proximity, *RES* responses to an interaction, *SMIL* smile, *FUNC* functional communication, *FP* functional play, *EMOG* emotional gestures, *DESCG* descriptive gesture, *OBJ* sharing object

In the group of participants with VIQ < 90, only 3 of the 6 participants-initiated interactions in session 2 (M = 2.67, SD = 2.08, Range [1–5]). As it can be seen in Figure 2, results show a significant relationship between deictic gestures (POINTG) that activated focal behavior (initiation of interaction) in 2 of the 6 participants (participant 15, Quadrant I, radius = 2.77, angle = 56.59°, p <0.01) (participant 10, Quadrant II, radius = 2.68, angle = 97.39°, p <0.01).

In session 10 a lower number of significant relationships was compared to session 2 and in a smaller number of participants in both groups (Figure 2). However, both eye contact (EC) (Quadrant II, radius = 3.55, SD_radius_= 0.19, angle = 95.41°, SD_angle_= 1.94, p < 0.01) and smile (SMIL) (Quadrant II, radius = 3.04, SD_radius_ = 0.93, angle = 96.87°, SD_angle_ = 4.06, p < 0.01) activated the initiation of social interactions in two participants from the VIQ > 90 group (participants 3 and 13). This type of relationship did not occur among participants in the VIQ < 90 group.

## Discussion and Conclusions

The main purpose of this study was to study social skills (“responding to interaction” and “initiating interaction”) and provide data quality about the possible changes in social behaviors in children with ASD who participated in an adapted social skills training program. We were interested in observing changes between the second and the last session of a social skills intervention program for children with ASD, through Polar Coordinate Analysis. Additionally, we aimed to evaluate differences in children according to their intelligence quotient. Regarding treatment, our purpose was to observe whether a brief intervention program could cause initial and little changes in social behavior adapted to our community context (Lerner & Mikami, 2012; Matthews et al., 2019).

Results show that, in general, our social skills intervention improved the quality of interactions but not the quantity, observing a diminution in the quantity of social behaviors in session 10 but improving the quality (more positive behaviors than in session 2).

Concerning methodology, mixed methods framework provides results that inform about the quality of the interactions established between the participants and also the existence or not of relational patterns. These micro-conducts and specific information were obtained through the rigorous observation in natural situations (Anguera, 2003; Portell et al., 2015) of the interactions during a spontaneous time-play (Deckers et al., 2016; McMahon et al., 2013; McMahon et al., 2013). According to several investigations (Koenig et al., 2010; Lerner & Mikami, 2012), questionnaires cannot provide that type of information, and this specific and detailed information can contribute to develop more specific interventions to improve social skills in children with autism. Many researches with brief interventions, expose that existed difficulties to conclude if their groups’ interventions were effective (Lerner & Mikami, 2012). With observational methodology, in our research, and inside *mixed methods* perspective, we could observe the micro-conducts that were at the beginning of the intervention and at the end, exposing the little differences before and after the intervention and getting more specificity in the process and evolution of each participant. These results were consistent with our previous study (Alcover et al., 2019), where we analyzed the evolution of social behaviors in five adolescents with ASD during an intervention of social skills. We concluded that the evolution and performance of social skills was different for each participant, observing better performance in some participants after the intervention.

In the present study, we aimed to study the influence of one more factor and used Polar Coordinate Analysis to detect possible changes associated with VIQ. Regarding relationship with verbal intelligence quotient (IQ), we found differences between sessions 2 and 10, both in social responses and initiations.

In our sample, we observed controversial but interesting results. Initiations of interactions were more complex and included more social components in participants with VIQ > 90 before and after intervention (initiations with social communication, eye contact, smile and use of gestures). Instead, the group with lower VIQ showed less patterns and low quality at the beginning. Regarding the response to an interaction, participants with VIQ < 90 showed a better improvement, specifically an activation of responses was observed through the behavior of looking without eye contact, indicating a low-quality level of interaction. According to Lebarton and Iverson research (2016), new significant pattern in these participants was observed, indicating an activation of the responses through conventional gestures. The results are highly variable, observing improvements in different areas in both groups; according with McMahon and colleagues (2013) that did not find a relationship between effectiveness of social skills training and IQ. However, other studies obtained better results in people with autism spectrum disorder and normal IQ rating (Gates et al., 2018). Children with higher intelligence coefficient have more social skills than children with lower IQ, but this does not imply that their evolution is better than others.

In relation to sex, we observed that two out of three females showed more patterns of responses to an interaction. There are many controversial aspects regarding this subject. Choque et al. (2017) observed more improvements in females than males. Our results did not include a significative number of both sexes, so we cannot affirm that girls perform better quality and quantity patterns than boys. Coinciding with another observational research, females performed more social interactions than males (McMahon et al., 2013; McMahon et al., 2013).

Generally, from results obtained in our study, we observed a better baseline of social responses in participants with higher VIQ. However, we observed new quantity and quality patterns after the intervention in participants with lower VIQ. With this research, we cannot conclude whether there are similar or common social behavior patterns between children with ASD, with a VIQ greater than 90, but we could better understand our participants, supporting and adapting our intervention with specific objectives to improve their impairments in social competence.

This knowledge could allow professionals to better understand the deficits and difficulties in communication and interaction that our participants tend to present and adapt their interventions, making them more efficient and beneficial for patients.

## Limitations

There are several limitations that should be considered for future researchers. Firstly, the most important limitation was the number of observations registered and codified. More observations are needed during the group and post-treatment to measure the effectiveness of the intervention. Having several observations during different times (at the beginning, in the middle, at the end and post-treatment) could contribute to have information about the evolution of the micro-conduct during the intervention (Alcover et al., 2019). Secondly, observers who coded the data were not blind to the study goals. Future research should consider the possibility that observers were totally blinded to avoid bias.Thirdly, our observation was only focused on social behaviors of each participant, but we did not include information about how one person’s behaviors are followed by other person’s reaction. This is an important limitation because it does not allow us to obtain information about how one participant responds to another person’s behaviors. Observation of dyadic interactions could provide information about other’s reactions. Finally, no control (waiting-list or traditional SSI; Lerner & Mikami, 2012; Marro et al., 2019) group was used, limiting interpretability of effects. We are a community hospital and we were limited by the clinical demand. However, we plan to perform further research with a control group (waiting list).

## Implications

Our findings show that mixed methodologies are useful to obtain information about patterns of micro-conducts that participants exhibit in social situations. Observational methodology can provide valuable data related to communication and social behaviors, complementing data obtained from other traditional methods, such as questionnaires. For example, observing new patterns of complex social behaviors can inform about the improvement of participants after the intervention. Moreover, the use of polar coordinate analysis offers information about the evolution of the changes of participants, session by session. This methodology allows us to observe the process of changement, and not only changes before and after an intervention. This information might be valuable to determine suitable modifications in the design of the intervention.

Furthermore, as aforementioned, future research could include information about dyadic interactions, which would allow professionals to understand sequences of behaviors between participants.

Moreover, based on the findings of this research, it is recommended to design social skills intervention groups for patients with ASD in community hospitals taking in account their VIQ level. We observed that both groups of patients (higher and lower VIQ) could learn and develop different social competences such as eye contact, gestures or social communication. In fact, each group had different needs and trajectory. It would be useful to design specific interventions considering the line base characteristics of each group (higher and lower VIQ) in order to obtain the maximum profit of the intervention.

## Supplementary Information

Below is the link to the electronic supplementary material.

Supplementary file1 (DOCX 35 kb)
