# Supplementary material for: Measuring Changes in Social Skills Throughout an Intervention Program for Children with ASD, Contributions from Polar Coordinate Analysis
Source: J Autism Dev Disord. 2022 Mar 12;53(6):2246–60. doi: 10.1007/s10803-022-05496-0 (PMC10229457; doi:10.1007/s10803-022-05496-0)
Supplement: Supplementary file 1 — Supplementary file1 (DOCX 35 kb) [file 10803_2022_5496_MOESM1_ESM.docx]

**Table S1**

*Results from the Descriptive Statistical Analysis of Session 2 and 10*

| **Dimensions**  Categories | **Session 2** | | | **Session 10** | | |  |  |  |  |
| --- | --- | --- | --- | --- | --- | --- | --- | --- | --- | --- |
| *Total of codified behaviors* | 1631 | | | 1393 | | |  |  |  |  |
| **Interaction type** | **Freq (%)** | **M**  **(SD)** | **Range** | **Freq**  **(%)** | **M**  **(SD)** | **Range** | **χ^2^(1)** | **t(20)** | ***p*** | ***d*** |
| Low-level interaction | 425  (94.65) | 20.24  (6.74) | 7—30 | 309 (85.12) | 14.71  (5.12) | 3—23 | 20.99*** | -3.77** | .001 | 0.82 |
| High level interaction | 24  (5.35) | 1.14  (1.39) | 0—4 | 46  (12.67) | 2.19  (3.03) | 0—11 | 13.68*** | 1.54 | .137 | 0.33 |
| Negative level interaction | - | - | - | 8  (2.2) | 0.38  (0.67) | 0—2 | - | - | - | - |
| *Total of interactions* | **449 (27.5%)** | | | **363 (26.06%)** | | | 21.815*** |  |  |  |
| **Social behavior** | | | |  | | |  |  |  |  |
| Responses | 362  (83) | 17.24  (5.69) | 8—28 | 267  (81.4) | 12.71  (4.7) | 3—20 | 0.079 | -3.49** | .002 | 0.762 |
| Initiations | 77  (18) | 3.67  (3.7) | 0—12 | 58  (17.69) | 2.76  (2.05) | 0—7 | 0 | -0.89 | .382 | 0.195 |
| Evitations | - | - | - | 3  (0.91) | 0.14  (0.21) | 0—1 | - | - | - | - |
| *Total social behaviors* | **439 (27%)** | | | **328 (23.55%)** | | | 4.50* |  |  |  |
| **Verbal communication** | | | |  |  | |  |  |  |  |
| Functional communication | 307  (96.5) | 14.62  (8.67) | 3—30 | 252  (92.99) | 12  (5.67) | 0—22 | 3.82 | -1.86 | .077 | 0.41 |
| Social verbal communication | 8  (2.5) | 0.38  (0.81) | 0—3 | 15  (5.53) | 0.71  (1.42) | 0—6 | 3.55 | 1.13 | .273 | 0.24 |
| Sharing experiences | - | - | - | 2  (0.74) | 0.09  (0.44) | 0—2 | - | - | - | - |
| Non-functional communication | 3  (0.94) | 0.14  (0.48) | 0—2 | 1  (0.37) | 0.05  (0.22) | 0—1 | - | - | - | - |
| Verbal aggressive communication | - | - | - | 1  (0.37) | 0.05  (0.22) | 0—1 | - | - | - | - |
| *Total verbal communication* | **318 (19.5%)** | | | **271 (19.45%)** | | | 0 |  |  |  |

| **Dimensions**  Categories | **Session 2** | | | **Session 10** | | |  | |  | |  |  |
| --- | --- | --- | --- | --- | --- | --- | --- | --- | --- | --- | --- | --- |
| **Facial expressions** | **Freq (%)** | **M**  **(SD)** | **Range** | **Freq (%)** | **M**  **(SD)** | **Range** | **χ^2^(1)** | | **t(20)** | | ***p*** | ***d*** |
| Smile | 8  (0.49) | 0.38  (0.67) | 0—2 | 26  (1.86) | 1.24  (2.02) | 0—7 | 12.79*** | | 1.88 | | .095 | 0.38 |
| **Looking** |  | | |  | | |  | |  | |  |  |
| Looking to an object or another situation | 148  (92.5) | 7.05  (6.45) | 0—18 | 131  (80.37) | 6.24  (4.29) | 0—13 | 10.1** | | -0.87 | | .397 | 0.19 |
| Eye contact | 11  (6.87) | 0.52  (0.98) | 0—3 | 29  (17.79) | 1.38  (2.27) | 0—9 | 8.87** | | 1.82 | | .082 | 0.39 |
| Looking without eye contact | 1  (.67) | 0.05  (0.22) | 0—1 | 3  (1.84) | 0.14  (0.48) | 0—2 | - | | - | | - | - |
| *Total looking* | **160 (9.8%)** | | | **163 (11.7%)** | | | 2.81 | |  | |  |  |
| **Gestures** |  | | |  | | |  | |  | |  |  |
| Pointing gestures | 111  (67.68) | 5.28  (4.45) | 0—14 | 87  (56.13) | 4.15  (3.38) | 0—10 | 4.52* | | -1.25 | | .227 | 027 |
| Emotional gestures | 23  (14) | 1.09  (1.2) | 0—4 | 26  (16.77) | 1.24  (1.49) | 0—5 | 0.46 | | 0.69 | | .497 | 0.15 |
| Conventional gestures | 15  (9.15) | 0.71  (1.15) | 0—4 | 24 (15.48) | 1.14  (1.14) | 0—3 | 2.98 | | 1.74 | | .097 | 0.38 |
| Affirmation or denial gesture | 9  (5.5) | 0.43  (0.75) | 0—2 | 8  (5.16) | 0.38  (0.97) | 0—4 | 0.02 | | -0.21 | | .833 | 0.04 |
| Descriptive gesture | 6  (3.65) | 0.29  (0.66) | 0—2 | 10  (6.45) | 0.48  (0.81) | 0—3 | 1.30 | 0.68 | | .504 | | 0.15 |
| *Total gestures* | **164 (10%)** | | | **155 (11.13%)** | | | 0.91 | |  | |  |  |

﻿*p < .05. **p < .01. ***p < .001
